# Flexible Strain Sensor Based on PVA/Tannic Acid/Lithium Chloride Ionically Conductive Hydrogel with Excellent Sensing and Good Adhesive Properties

**DOI:** 10.3390/s25154765

**Published:** 2025-08-01

**Authors:** Xuanyu Pan, Hongyuan Zhu, Fufei Qin, Mingxing Jing, Han Wu, Zhuangzhi Sun

**Affiliations:** Province Key Laboratory of Forestry Intelligent Equipment Engineering, College of Mechanical and Electrical Engineering, Northeast Forestry University, Harbin 150040, China

**Keywords:** polyvinyl alcohol, tannins, ion-conducting hydrogels, strain sensors

## Abstract

Ion-conductive-hydrogel strain sensors demonstrate broad application prospects in the fields of flexible sensing and bioelectric signal monitoring due to their excellent skin conformability and efficient signal transmission characteristics. However, traditional preparation methods face significant challenges in enhancing adhesion strength, conductivity, and mechanical stability. To address this issue, this study employed a freeze–thaw cycling method, using polyvinyl alcohol (PVA) as the matrix material, tannic acid (TA) as the adhesion reinforcement material, and lithium chloride (LiCl) as the conductive medium, successfully developing an ion-conductive hydrogel with superior comprehensive performance. Experimental data confirm that the PVA-TA-0.5/LiCl-1 hydrogel achieves optimal levels of adhesion strength (2.32 kPa on pigskin) and conductivity (0.64 S/m), while also exhibiting good tensile strength (0.1 MPa). Therefore, this hydrogel shows great potential for use in strain sensors, demonstrating excellent sensitivity (GF = 1.15), reliable operational stability, as the Δ*R/R*_0_ signal remains virtually unchanged after 2500 cycles of stretching, and outstanding strain sensing and electromyographic signal acquisition capabilities, fully highlighting its practical value in the fields of flexible sensing and bioelectric monitoring.

## 1. Introduction

In recent years, the demand for human motion monitoring technology in the field of wearable electronics has continued to grow, particularly in the areas of health management [1], rehabilitation medicine [2], and human–computer interaction [3]. Traditional strain sensors are prepared by composite elastic substrates using materials such as conductive polymers [4,5], metals [6], carbon black [7], and graphene [8], which are characterized by high sensitivity but generally suffer from excessive rigidity (tensile ratio < 100%) and insufficient wearing comfort, mainly stemming from the significant modulus difference between the abovementioned material and skin tissue. The research focus in the field of flexible strain sensors has shifted to hydrogel and elastomer material systems, whose modulus (0.1–1 MPa) is highly compatible with biological tissues and whose tensile rate is generally >100% [9,10,11]. For example, flexible strain sensors prepared by laser irradiation using silicone as the elastic base and MWCNT as the conductive material have excellent stretchability (725%) and extremely short response times (1 ms) [12]. Therefore, hydrogels for sensors usually are required to have excellent tensile, conductive, and adhesion properties [13,14,15]. Among them, the surface adhesion ability ensures that the hydrogel can be closely adhered to the detection surface, thus enhancing the sensing accuracy [16]; the excellent tensile properties enable it to withstand persistent mechanical deformation [17]; and the conductive properties are the key elements for realizing the conversion of mechanical signals to electrical signals [18]. However, realizing the synergistic optimization of the three remains the focus of current technological research. Agniva Dutta’s research group used a one-pot synthesis process to prepare an all-physical crosslinked metal hydrogel, which possessed a tensile strength of 6.77 MPa and an elastic modulus of 155 MPa [19]. The polyacrylamide bi-network hydrogels developed by Ou’s team exhibited an elongation at break of more than 1500% and a tensile strength of 581 kPa, but these materials were limited by their own insufficient adhesive properties, and still needed to rely on external adhesive materials to assist fixation in the device assembly process [20]. Existing research on strengthening mechanical properties primarily focuses on optimizing the cross-linking agent system; however, it is challenging to account for electrical conductivity. The catechol lignin/liquid metal composite hydrogel developed by Zhao’s team achieved an interfacial adhesion strength of 16.23 kPa through structural modification, but only attained a conductivity of 0.24 S/m, which was insufficient to support the demand for precision signal monitoring [21]. The agar/NaCl/PAM dual-network hydrogel developed by Hou’s team had a high tensile strength and high elongation at break, with a tensile strength of 400 kPa [22]. However, there are still obvious shortcomings in electrical conductivity (0.4 S/m) and sensitivity (GF = 0.5), which restrict the practical application value.

In this study, a three-dimensional porous network was successfully constructed by combining the interfacial adhesion function of tannic acid with the ion-transport property of Li^+^/Cl^−^ by using poly (vinyl alcohol) as a substrate. Among the groups of tannic acid, the polyphenolic hydroxyl group realized interfacial bonding with biological tissues through hydrogen bonding, and Li^+^/Cl^−^ ions mainly took on the function of charge-transport carriers. The comprehensive performance of the optimized PVA-TA-0.5/LiCl-1 hydrogel sensor was significantly improved: the adhesion strength to porcine skin tissue reached 2.32 kPa, the electrical conductivity was enhanced to 0.064 S/m, and the tensile strength was stably maintained at 0.11 MPa. The material exhibited excellent sensitivity characteristics (GF = 1.15) and stable sensing performance. Under different tensile strain conditions and frequencies, the resistance response of the hydrogel was stable, and the output signal remained stable after 2500 cycles of tensile testing. In the practical application test, the hydrogel-based sensor could not only accurately recognize the resistance change in finger bending in the range of 0–90°, but also the impedance value of the skin contact interface in the wide frequency range of 10^0^–10^5^ Hz was significantly lower than that of traditional Ag/AgCl gel electrode products. Meanwhile, it could reliably collect clear electromyographic signals with different fist shaking strengths, which strongly verified the practical application of this ionic hydrogel in flexible sensing devices and bioelectrical signal monitoring systems.

## 2. Materials and Methods

### 2.1. Materials

The chemical reagents used in the experiments included polyvinyl alcohol (PVA, alcoholysis degree: 99%, Mw: 74,800–79,200; Sinopharm Chemical Reagent Co., Ltd., Shanghai, China), tannic acid (TA, purity: 98%; Shanghai McLean Biochemical Technology Co., Ltd., Shanghai, China), and anhydrous lithium chloride (LiCl, purity: 99.5%; Shanghai Zhanyun Chemical Co., Ltd., Shanghai, China). For the experimental materials, research-grade pigskin, a steel plate, and an acrylic plate were purchased through online channels. The pigskin was sterilized with ethanol and used exclusively for the adhesion strength test. Deionized water was used as the medium in the experimental process, which was prepared and used on-site.

### 2.2. Preparation of Hydrogel

**Figure 1:**
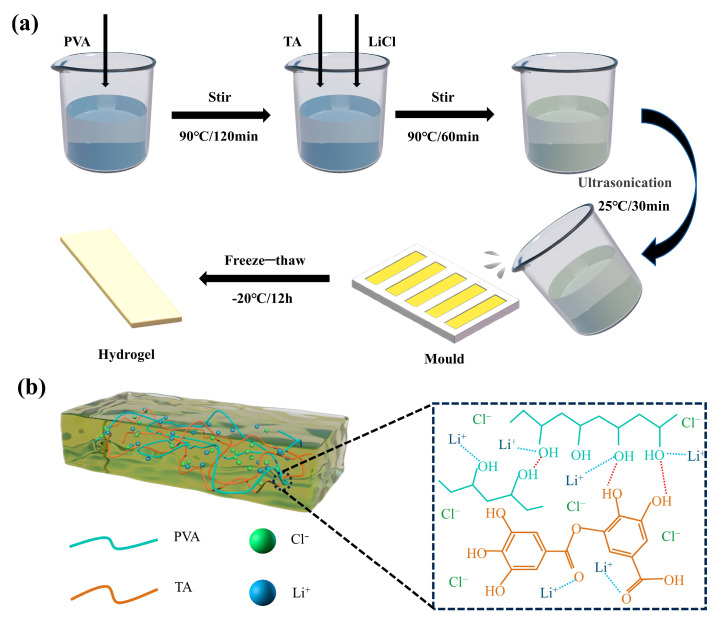
(**a**) Preparation procedure of PVA-TA/LiCl hydrogel. (**b**) Schematic diagram of PVA-TA/LiCl hydrogels showing PVA-TA hydrogen bonding and Li^+^ coordination.

The preparation process of the PVA-TA/LiCl hydrogel is shown in Figure 1a. First, 5 g of polyvinyl alcohol (PVA) was added to 45 mL of deionized water and continuously stirred at a constant temperature of 90 °C for 2 h to prepare a PVA precursor solution with a concentration of 10 wt%. Subsequently, x g of tannic acid (TA) with y g of lithium chloride (LiCl) were added sequentially, and ultrasonic degassing was carried out after continued stirring for 1 h. The mixed solution was poured into a laboratory-made double dumbbell or rectangular mold and then frozen at −20 °C for 12 h and thawed at room temperature for 1 h to complete the cross-linking process. For easy differentiation, the fabricated ion-conductive hydrogels were named PVA-TA-x/LiCl-y (x and y corresponded to the feeding mass of TA monomer and LiCl, respectively). The cross-linking state inside the PVA-TA/LiCl hydrogel is shown in Figure 1b. During the freeze–thaw process, the crystallization of PVA and phase separation caused the mixed solution of PVA/TA/LiCl to become a gel, where TA formed strong hydrogen bond interactions with PVA and the Li coordinated with the hydroxyl groups on PVA and the ketone groups on TA [23,24,25,26]. Based on the single-variable principle, a series of PVA-TA-x samples without LiCl and with varying amounts of TA monomer were prepared using the same process. For comparison, pure PVA hydrogels without TA and LiCl were also prepared according to the abovementioned procedures.

### 2.3. Characterization

The micro-morphologies of the hydrogel samples were observed using a scanning electron microscope (GeminiSEM 300, Carl Zeiss, Oberkochen, Germany). Before the tests, the samples were freeze-dried in a vacuum freeze-dryer (SCIENTZ-10N/A, Ningbo Xinzhi Biotechnology Co., Ltd., Ningbo, China) for 24 h, and then coated with conductive gold to observe their microstructure. The Fourier transform infrared spectra of all the hydrogel samples were measured using a Nicolet iS20 Fourier transform infrared spectrometer (Thermo Fisher Scientific, Waltham, MA, USA). The scanning range was from 4000 to 800 cm^–1^. All the samples were freeze-dried before testing.

### 2.4. Mechanical Properties Testing of Hydrogel

(1) Adhesive properties

The hydrogel specimens were accurately cut into standard sizes of 7 mm × 7 mm × 1 mm (length × width × height). The pigskin, acrylic board, and steel plate were selected as adhesive substrate materials. These substrate materials were cut into standard sizes of 60 mm × 10 mm × 1 mm (length × width × height), and then fixed at both ends using a tensile testing machine (DR-507A, Dongguan Dongri Instrument Co., Ltd., Dongguan, China). The cut hydrogels were placed between the substrate materials and then manually pre-pressurized for 5 min. The shear force–displacement curve was determined at a loading rate of 10 mm/min. Its adhesion shear strength value was calculated according to Equation (1):

(1) $$\tau =\frac{F}{A}$$

where τ is the adhesion shear strength, *F* is the maximum load at shear breakage, and *A* is the bond area.

(2) Tensile properties

The tensile properties of hydrogel specimens were tested using a universal tensile testing machine with a loading rate of 50 mm/min. The specimens were prepared using a double dumbbell-shaped tensile notch mold developed by the laboratory. At least three repetitions of the test were carried out for each group.

### 2.5. Conductivity Measurements

The conductive hydrogel samples were cut into 60 mm × 5 mm × 3 mm strip sizes. Two platinum electrodes were inserted 40 mm apart at both ends of the samples and connected to an electrostatic meter (Keithley 6514, Beijing Hanlei Science and Technology Co., Ltd., Beijing, China) for resistance measurement. All the samples were repeated three times or more. We calculated the conductivity value according to Equation (2):

(2) $$\sigma =\frac{L}{AR}$$

where σ is the conductivity of the sample, *L* is the test length of the sample, *A* is the test cross-sectional area of the sample, and *R* is the resistance of the sample.

### 2.6. Sensitivity and Linearity of Hydrogel

The sensitivity of the conductive hydrogel was characterized by the gauge factor (GF). The samples were prepared with the same dimensions as the tensile specimens, and the platinum electrodes were inserted at both ends to connect the wires and then fixed in a tensile testing machine. The other end of the wire was connected to a multimeter electrode and stretched at a rate of 50 mm/min, and the resistance change was continuously monitored by a multimeter (NPLC = 1). Linearity was determined by linear fitting of the resistance change–strain scatter plot, and the relative resistance change and GF were calculated by Equations (3) and (4), respectively:

(3) $$\frac{\Delta R}{R_{0}}=\frac{R-R_{0}}{R_{0}}$$

where Δ*R/R*_0_ is the relative resistance change in the conductive hydrogel, *R* is the real-time resistance of the conductive hydrogel, and *R*_0_ is the initial resistance of the conductive hydrogel.

(4) $$GF=\frac{\Delta R}{\varepsilon R_{0}}$$

where *GF* is the gauge factor of the conductive hydrogel, Δ*R* is the resistance change in the conductive hydrogel, *ε* is the relative change in elongation of the conductive hydrogel ((*l* − l_0_)/*l*_0_), and *R*_0_ is the initial resistance of the conductive hydrogel.

### 2.7. Sensing Performance of Hydrogel

The specimen was connected to the wire and fixed in the tensile testing machine according to the above sensitivity-testing method. Subsequently, the conductive hydrogel was continuously stretched five times at different tensile strains. The conductive hydrogel was subjected to cyclic tensile testing at different frequencies for 200 s. The conductive hydrogel was subjected to continuous cyclic tensile testing at a fixed tensile strain of 120% for 2500 cycles, with a cyclic tensile rate of 500 mm/min. During the above testing process, the resistance value of the conductive hydrogel was continuously recorded using an electrometer. The NPLC value of the electrometer was set to 1.

### 2.8. Strain Sensing and Electromyographic (EMG) Signal Acquisition Functional Applications

The ionized conductive hydrogel was applied to the finger and wrist joints of the subjects. An electrometer instantly acquired the electrical signals at different bending angles of the joints, and the stabilized posture was maintained for 5 s at each angle. In the EMG signal acquisition experiments, subjects repeated the fist-clenching action with three levels of fist-clenching strength: low, medium, and high. The corresponding electrical signals output from the hydrogel electrodes were collected by EMG sensors (HKJ-15C, Hefei Huake Electronic Technology Research Institute, Hefei, China). The variation in the signal amplitude with the strength of the fist-clenching was observed. All EMG signal data were filtered by MATLAB R2024a for analysis of sensing capabilities. The frequency impedance spectra were recorded using an electrochemical workstation (CS350H, Wuhan Koste Instrument Co., Wuhan, China). Finally, the interfacial contact impedance of PVA-TA-0.5/LiCl-1 and commercial gel electrodes was compared in order to comprehensively evaluate the performance of the hydrogel in human motion monitoring and EMG signal detection.

### 2.9. Water Retention Performance of Hydrogel

For the water retention test, all freshly prepared hydrogels were placed at 25 °C and weighed every 12 h. The water retention capacity of these hydrogels at different times was calculated by the following Equation (5):

*Water retention ratio* (%) = (*W_t_*/*W*_0_) × 100% (5)

where *W*_0_ and *W_t_* are the initial weights and the weights of the samples at specific times, respectively.

## 3. Results

### 3.1. Mechanical Properties of Hydrogel

**Figure 2:**
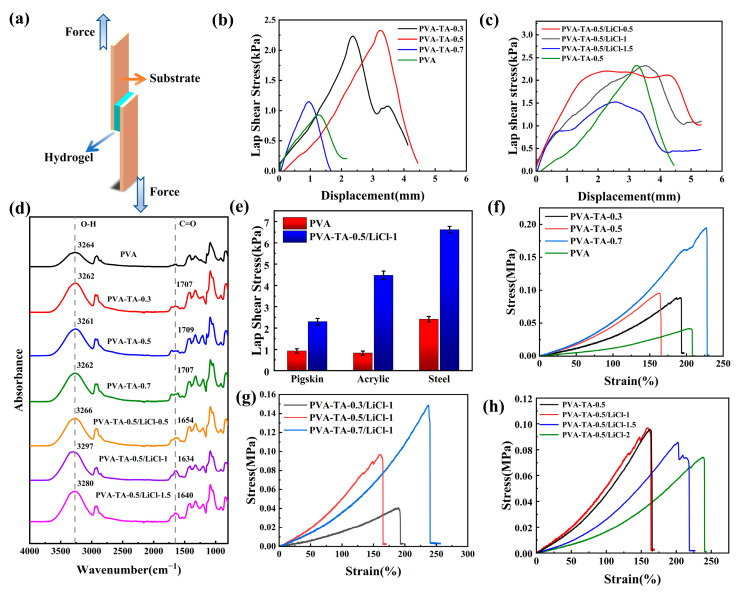
(**a**) Schematic of adhesion lap shear experiment. (**b**) Shear stress–displacement curves for different TA contents in PVA/TA hydrogels. (**c**) Shear stress–displacement curves for different LiCl contents in PVA/TA/LiCl hydrogels. (**d**) Infrared spectral curves of PVA, PVA-TA, and PVA-TA/LiCl hydrogels. (**e**) Comparison of effect of PVA and PVA-TA-0.5/LiCl-1 hydrogel on adhesion strength of pigskin, acrylic, and steel. (**f**) Tensile stress–strain curves in PVA/TA hydrogels with different TA contents, and for (**g**) PVA/TA/LiCl hydrogels. (**h**) Tensile stress–strain curves for different LiCl contents in PVA/TA/LiCl hydrogels.

The schematic of the adhesion shear test is shown in Figure 2a. As shown in Figure 2b, it could be clearly seen that the weight of TA had a significant effect on the adhesion properties of the PVA-TA hydrogels. When the weight of TA was 0 g, 0.3 g, 0.5 g, and 0.7 g, the adhesive strengths of the hydrogel were 0.92 kPa, 1.15 kPa, 2.32 kPa, and 2.23 kPa, respectively. Consequently, as the weight of TA increased, the adhesion strength of the interface between the PVA-TA hydrogels and the pigskin first gradually increased and then decreased. When the weight of the TA reached 0.5 g, its adhesion strength was 1.5 times that of PVA hydrogel. As shown in Figure 2d, the hydroxyl (–OH) vibration peak of the PVA hydrogel was presented at 3264 cm^−1^ [27]. When different weights of TA were added, the –OH vibration peak of PVA-TA hydrogels were red-shifted compared to the PVA hydrogel, which indicated the formation of a strong intermolecular hydrogen bonding interaction between the hydroxyl group of PVA and the phenolic hydroxyl group of TA [24]. It was worth noting that the red-shift was greatest when TA was 0.5 g compared to 0.3 g and 0.7 g, meaning that when TA was 0.5 g, there was the greatest hydrogen bonding interaction between TA and PVA. Therefore, the multiple hydrogen bonding network inside the PVA-TA-0.5 hydrogel and the hydrogen bonding interactions between its –OH groups and the N and O elements on the surface of the substrate synergistically enhanced the adhesion properties [28]. However, it was noted that the mixed PVA/TA solution could not be gelled due to the pre-cross-linking generation between them when the weight of the TA reached 1 g. Although the high-density catechol groups in TA could enhance adhesion and mechanical strength [29], excessive addition could induce excessive cross-linking of PVA and inhibit the formation of the gel network [30]. To ensure the adhesion of the hydrogels, the weight of TA was set to 0.5 g for the following experiments.

As shown in Figure 2c, the adhesion properties of the hydrogels remained almost unchanged when the weight of LiCl was 0 g, 0.5 g, and 1 g. However, it was decreased when the weight of LiCl was 1.5 g. This indicated that the addition of a small amount of LiCl had almost no effect on adhesion properties. In contrast, when the weight of LiCl was 1.5 g, its adhesion strength to the pigskin substrate decreased from the initial 2.32 kPa to 1.52 kPa (34.8% decrease), which was attributed to the ionic solvation effect due to the high concentration of Li^+^, resulting in the dissociation of the dynamic hydrogen bonding network. As shown in Figure 2d, the hydroxyl (–OH) vibrational peak of the PVA-TA-0.5/LiCl hydrogels was blue-shifted compared to the PVA and PVA-TA hydrogels, which suggested that Li^+^ in the hydrogel weakened the hydrogen bonding structure between the PVA chains or between the PVA and the TA [25]. As a result, the three-dimensional structure in the PVA-TA-0.5/LiCl-1 hydrogel network became sparse, which in turn caused a decrease in the adhesion properties. It was interesting to note that the C=O vibration peak of the PVA-TA-0.5 hydrogel was presented at 1709 cm^−1^ [31]. When LiCl was added, the C=O vibration peak of the PVA-TA-0.5/LiCl hydrogel was red-shifted, with an increase in peak intensity compared to PVA-TA-0.5 hydrogel, which was attributed to the formation of a coordination bond interaction between the Li^+^ and the ketone group of TA [26]. In summary, to achieve both optimal adhesion properties and excellent ionic conductivity in the hydrogel, it was preliminarily determined that the optimal adhesion effect could be realized for the PVA-TA-0.5/LiCl-1 hydrogel when the weight of TA was 0.5 g and the weight of LiCl was 1 g. In addition, as shown in Figure 2e, the adhesion strength of the PVA-TA-0.5/LiCl-1 hydrogel on acrylic and metal substrates was significantly improved compared with that of PVA hydrogel, both reaching 4.47 kPa and 6.61 kPa, respectively, which reflected the good generalization of the adhesion performance of the PVA-TA-0.5/LiCl-1 hydrogel for different substrates.

The tensile properties of the PVA-TA hydrogels are exhibited in Figure 2f, where the tensile strength of the PVA-TA hydrogels gradually increased with the increase in TA weight, which was attributed to the formation of strong intermolecular hydrogen bonding interactions between the hydroxyl groups of PVA and the phenolic hydroxyl groups of TA. When the weight of TA was 0.5 g, the tensile strength of the PVA-TA-0.5 hydrogel reached 0.1 MPa, which was 2.5 times that of the PVA hydrogel. Furthermore, when the LiCl with a weight of 1 g was added to PVA-TA-0.5 hydrogel, the tensile properties of the PVA-TA/LiCl hydrogels decreased overall compared to the corresponding PVA-TA hydrogels, as shown in Figure 2g. This was due to the weakening of the hydrogen bonding structure between the PVA chains or between PVA and TA by Li^+^. It is worth noting that the tensile strength reached its peak when the weight of TA was 0.7 g, indicating that the hydrogen bond density and network structure had achieved an optimal balance. However, the tensile strengths of hydrogels with excessively high TA weights were reduced due to uneven cross-linking [32]. The effect of the weight of LiCl on the PVA-TA-0.5 hydrogel was demonstrated in Figure 2h, where the tensile properties of the hydrogels gradually decreased as the weight of LiCl rose. Moreover, when the weight of LiCl was 0.5 g and 1 g, the tensile properties of the corresponding PVA-TA-0.5/LiCl-1 hydrogel and PVA-TA-0.5 were almost the same. The tensile strength and strain at break of the PVA-TA-0.5/LiCl-1 hydrogel were 0.1 MPa and 164%, respectively. Consequently, the good tensile and mechanical properties provide a good basis for this hydrogel’s use as a strain sensor substrate. Based on the need for high electrical conductivity, the PVA-TA-0.5/LiCl-1 hydrogel was selected for subsequent tests. Cyclic tensile experiments were conducted to evaluate the fatigue resistance of the PVA-TA-0.5/LiCl-1 hydrogels. It could be seen from Figure S1 that under tensile cycling at the 70% strain, the dissipated energy of the hydrogel was low and did not change significantly over 10 cycles, indicating that the hydrogel had excellent self-recovery properties over the human motion range [33].

### 3.2. Conductivity, Sensitivity, and Sensing Performance of Hydrogels

**Figure 3:**
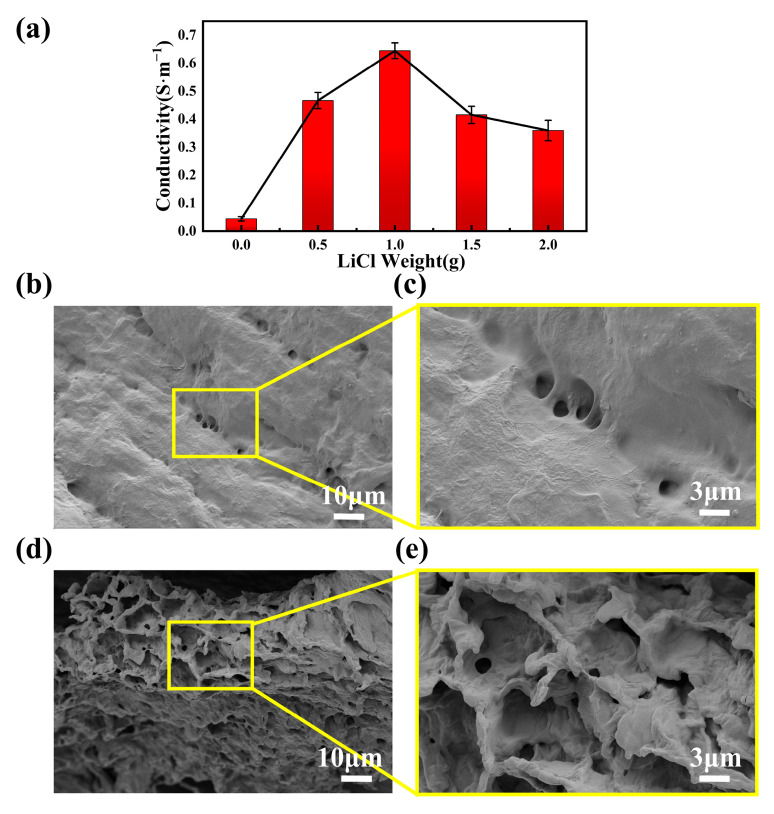
(**a**) Histogram of the effect of different LiCl contents on electrical conductivity. (**b**) SEM image and local magnification of PVA hydrogel (**c**). (**d**) SEM image and local magnification of PVA-TA-0.5/LiCl-1 hydrogel (**e**).

As shown in Figure 3a, with an increase in LiCl weight, the electrical conductivities of the PVA-TA/LiCl hydrogels first increased gradually and then decreased. When the weight of LiCl reached 1 g, the conductivity of the PVA-TA-0.5/LiCl-1 hydrogel reached 0.64 S/m, which was 16 times higher than that of the PVA-TA-0.5 hydrogel. This is because LiCl was ionized inside the hydrogel to generate a large amount of Li^+^ and Cl^−^, which effectively increased the free ion concentration. However, the electrical conductivity of the hydrogel was reduced when the weight of LiCl was 1.5 g. This was attributed to the fact that the excess Li^+^ and Cl^−^ triggered the Hoffmeister effect through solvation, which weakened the cross-linking strength of the polymer chains through the formation of coagulation between the PVA chains, resulting in the structural collapse of the ion-transporting channels [34,35]. The microscopic morphologies of the PVA hydrogel and PVA-TA-0.5/LiCl-1 hydrogel are presented in Figure 3b–e. In Figure 3b,c, the PVA hydrogel exhibits a dense three-dimensional network structure. In contrast, as shown in Figure 3d,e, the PVA-TA-0.5/LiCl-1 hydrogel showed a three-dimensional network structure with a larger pore size. The larger porous network provided an efficient transport channel for Li^+^, thereby improving the ionic conductivity of the hydrogel.

According to the analysis of the above experimental results, it could be concluded that the prepared conductive hydrogel showed optimal adhesive, conductive, and tensile properties when the weights of TA and LiCl were 0.5 g and 1 g, respectively. Therefore, the PVA-TA-0.5/LiCl-1 hydrogel was finally selected as the research object for the sensing tests.

**Figure 4:**
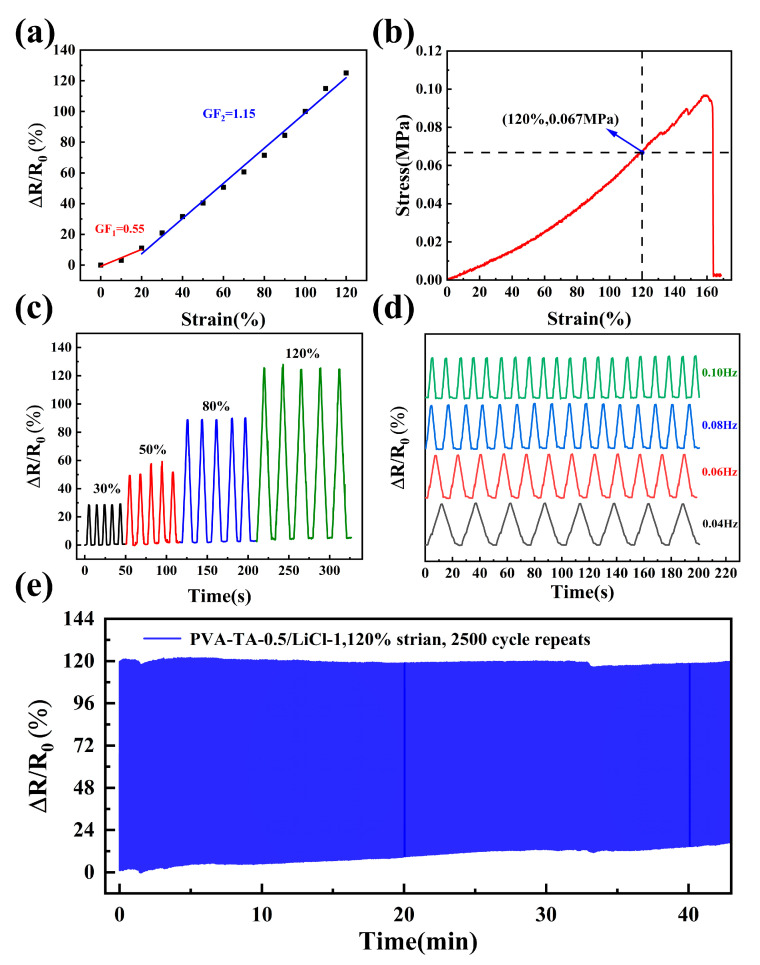
(**a**) Linear fitting curve between relative resistance change and tensile strain of PVA-TA-0.5/LiCl-1 hydrogel. (**b**) Tensile stress–strain curve of PVA-TA-0.5/LiCl-1 hydrogel. (**c**) Relative resistance change in PVA-TA-0.5/LiCl-1 hydrogel after 5 consecutive cycles of stretching at different strains. (**d**) Relative resistance changes in PVA-TA-0.5/LiCl-1 hydrogel at different frequencies. (**e**) Relative resistance of PVA-TA-0.5/LiCl-1 hydrogel stretched under 120% strain for 2500 consecutive cycles.

Sensitivity and linearity are key parameters for sensing in a flexible strain sensor. Sensitivity was assessed by gauge factor (GF) and linearity was determined by the signal detection accuracy [36]. By the linear fitting curves displayed in Figure 4a, the GF was calculated to be 0.55 and 1.15 in the 0–20% and 20–120% strain range, respectively, which was comparable to that of most of the reported hydrogel-based strain sensors and sufficient for monitoring multi-strain human body movements (0–75%) [33,37,38]. Furthermore, the linear correlation coefficients of the fitted equations obtained by calculation were 0.94 and 0.99, respectively, both greater than 0.99, indicating that the PVA-TA-0.5/LiCl-1 hydrogel had excellent sensitivity and linearity. Nevertheless, the resistance increased dramatically when the strain of the PVA-TA-0.5/LiCl-1 hydrogel exceeded 120%, indicating that the sensor had been destroyed [39]. Therefore, the effective working strain range of PVA-TA-0.5/LiCl-1 hydrogel was 0–120%. As shown in Figure 4b, the effective stress range of the PVA-TA-0.5/LiCl-1 hydrogel was 0–0.067 MPa. Combined with Figure 2g, it could be concluded that the mechanical properties of the PVA-TA-0.5/LiCl-1 hydrogel were fully capable of meeting the requirements for continuous detection under its effective sensing strain range.

The electrical resistance response of the PVA-TA-0.5/LiCl-1 hydrogel in five cyclic stretch–release tests at different tensile strains was illustrated in Figure 4c. When the hydrogel was constantly stretched to 30%, 50%, 80%, and 120% strain, the Δ*R/R*_0_ increased to 28%, 49%, 88%, and 125%, respectively, indicating that the PVA-TA-0.5/LiCl-1 hydrogel could sense different strain magnitudes. Figure 4d depicts that the output Δ*R/R*_0_ signals by cyclic stretching could be kept relatively stable at strain frequencies of 0.04 Hz, 0.06 Hz, 0.08 Hz, and 0.1 Hz. Meanwhile, Figure 4e indicates that the output Δ*R/R*_0_ signal of the PVA-TA-0.5/LiCl-1 hydrogel remained highly stable after undergoing 2500 consecutive stretch and release cycles at 120% strain. Notably, although the output Δ*R/R*_0_ signal exhibited slight fluctuations due to mechanical hysteresis during the 2500 stretch cycles, it was relatively stable overall, and the hydrogel also maintained a good conductivity of 0.58 S/m (Figure S2) [40]. As shown in Figure S3, the PVA-TA-0.5/LiCl-1 hydrogel showed low hysteresis (4.36%) in the comparison of loading and unloading processes, which was comparable to that of most of the reported hydrogel-based strain sensors (0–10%) [41,42]. Consequently, the PVA-TA-0.5/LiCl-1 hydrogel demonstrated reliable electrical signal output at 120% strain, confirming its superior sensing performance.

### 3.3. Application of PVA-TA-0.5/LiCl-1 Hydrogel Strain Sensors

**Figure 5:**
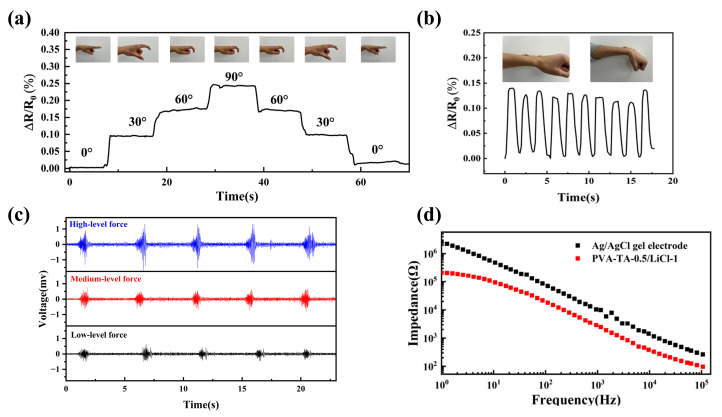
Application of PVA-TA-0.5/LiCl-1 hydrogel. Detection of relative resistance changes for joint movements: (**a**) finger flexion, (**b**) wrist. (**c**) EMG signals when holding grip at different levels of force. (**d**) Impedance spectra of commercial Ag/AgCl gel electrode and PVA-TA-0.5/LiCl-1 skin interface.

Based on its excellent adhesion, ductility, electrical conductivity, and sensing properties, the PVA-TA-0.5/LiCl-1 hydrogel could be used as a flexible strain sensor substrate to realize the accurate monitoring of human movement status and related EMG signals. Figure 5a and Movie S1 display that the different bending angles of the finger could be precisely reflected through the Δ*R/R*_0_. When the finger was bent to different angles at 30°, 60°, and 90°, the resistance increased stepwise to specific values of 9.5%, 17.5%, and 24.3% and remained constant. As shown in Figure 5b and Movie S2, the PVA-TA-0.5/LiCl-1 hydrogel could also clearly sense wrist bending, and the output Δ*R/R*_0_ signal remained stable overall. As shown in Figure 5c and Movie S3, when the subject repeated the fist-clenching action with three kinds of strengths—light, medium, and strong—the amplitudes of the EMG signal were, respectively, 0.4 mV, 0.6 mV, and 1.3 mV, and the corresponding signal-to-noise ratios were, respectively, 22.1 dB, 22.4 dB, and 35.5 dB. This indicated that the PVA-TA-0.5/LiCl-1 hydrogel was able to capture EMG signals with well-defined amplitudes and stable waveforms successfully. During limb movement, changes in muscle state caused surface fluctuations in the arm, leading to relative movement between the muscle, skin, and electrodes. This relative movement could create motion artifacts in the electromyographic signals, resulting in baseline drift [43]. In order to better present stable signal changes, high-pass filtering was applied to EMG signals with baseline drift, which led to the difference between Figure 5c and Movie S3. Figure 5d clarifies that the interface impedance between the PVA-TA-0.5/LiCl-1 hydrogel and skin was smaller than that of the commercial Ag/AgCl gel electrode in the frequency range of 10^0^–10^5^ Hz, which almost covers the range of EMG bioelectrical signals [44]. Additionally, all the constituent materials in the PVA-TA-0.5/LiCl-1 hydrogel exhibited excellent biocompatibility [28,45,46]. Furthermore, as shown in Figure S4, the PVA-TA-0.5/LiCl-1 hydrogel did not cause inflammation on the skin within 3 h, further indicating the excellent biocompatibility of the PVA-TA-0.5/LiCl-1 hydrogel. On the other hand, to broaden the applications and improve their service life in practical application, ionic conductive hydrogels with long-term moisturizing properties are essential [13]. As shown in Figure S5, after being placed at 25 °C for 96 h, the PVA-TA-0.5/LiCl-1 hydrogel had a water content of 21%, which was better than that of the PVA-TA-0.5 hydrogel. This was due to LiCl reducing water loss in the hydrogel through ionic solvation [47]. Therefore, the addition of LiCl could enable the PVA-TA-0.5/LiCl-1 hydrogel to have good conductivity while improving its durability for long-term work. Consequently, the PVA-TA-0.5/LiCl-1 hydrogel has great potential for application in the field of strain sensing and EMG signal acquisition.

## 4. Conclusions

In summary, a strain sensor based on the PVA/TA/LiCl hydrogel has been successfully constructed in this study, revealing the mechanism of multiple interactions between components that regulate material properties. The experimental results showed that when the additions of TA and LiCl were 0.5 g and 1 g, respectively, the PVA-TA-0.5/LiCl-1 hydrogel exhibited excellent comprehensive performance, with an adhesion strength of 2.32 kPa, an electrical conductivity of 0.64 S/m, and a tensile strength of 0.1 MPa. In terms of sensing performance, the PVA-TA-0.5/LiCl-1 hydrogel exhibited excellent stability and frequency response characteristics, maintaining a stable electrical signal output under various strain amplitude and frequency conditions. In the application scenario of strain sensing and EMG signal acquisition, the hydrogel could not only monitor the change in finger bending angle (0–90°) with high precision and maintain the stability of the electrical signal, but its skin contact impedance in the frequency range of 1–10^5^ Hz was also much lower than that of commercial Ag/AgCl gel electrodes, and it successfully captured EMG signals with a clear amplitude and regular waveforms, fully demonstrating that the material was suitable for joint motion monitoring and EMG signal acquisition. In addition, the PVA-TA-0.5/LiCl-1 conductive hydrogel developed in this study provided a new idea for the design and application of flexible strain sensors, which are expected to play an important role in the future wearable devices, human health monitoring, and human–computer interaction.

## Data Availability

The data that support the plots within this paper and other findings of this study are presented in the main article and the Supplementary Materials. Additional data related to this paper may be requested from the corresponding authors upon reasonable request.

## Supplementary Materials

The following supporting information can be downloaded at: https://www.mdpi.com/article/10.3390/s25154765/s1, Movie S1: Sensing performance of PVA-TA-0.5/LiCl-1 hydrogel at different angles of hand knuckle bending; Movie S2: Sensing performance of PVA-TA-0.5/LiCl-1 hydrogel on hand bowl joint bending; Movie S3: Application of PVA-TA-0.5/LiCl-1 hydrogel for EMG signal acquisition. Figure S1: Ten cyclic tensile curves of PVA-TA-0.5/LiCl-1 hydrogel at 70% strain; Figure S2: Conductivity of PVA-TA-0.5/LiCl-1 hydrogel stretched to 120% strain at different cycles; Figure S3: Relative resistance change in PVA-TA-0.5/LiCl-1 hydrogel strain sensors under 120% strain; Figure S4: PVA-TA-0.5/LiCl-1 hydrogel did not cause inflammation on skin after 3 h; Figure S5: Water retention performance of PVA-TA-0.5/LiCl-1 hydrogel and PVA-TA-0.5 hydrogel.
