# Supplementary material for: Flexible Strain Sensor Based on PVA/Tannic Acid/Lithium Chloride Ionically Conductive Hydrogel with Excellent Sensing and Good Adhesive Properties
Source: Sensors (Basel). 2025 Aug 1;25(15):4765. doi: 10.3390/s25154765 (PMC12349623; doi:10.3390/s25154765)
Supplement: Supplementary file 1 [file sensors-25-04765-s001.zip › sensors-3768360.pdf]

Supplementary Materials for

Flexible Strain Sensor Based on PVA/Tannic Acid/Lithium Chloride Ionically Conductive Hydrogel with Excellent Sensing and Good Adhesive Properties

Xuanyu Pan†, Hongyuan Zhu†, Fufei Qin, MingXing Jing, Han Wu* and Zhuangzhi Sun*

Province Key Laboratory of Forestry Intelligent Equipment Engineering, College of Mechanical and Electrical Engineering, Northeast Forestry University, Harbin 150000, China; 19305932833@163.com (X.P.); 2023111641@nefu.edu.cn (H.Z.); qinfufei@nefu.edu.cn (F.Q.); jingmingxing@nefu.edu.cn (M.J.)

* Correspondence: whan@nefu.edu.cn (H.W.); sunzhuangzhi@nefu.edu.cn (Z.S.)

† These authors contributed equally to this work.

Brief description of what thisfile includes:

Figure S1. 10 cyclic tensile curves of the PVA-TA-0.5/LiCl-1 hydrogel at the 70% strain.

Figure S2. Conductivity of PVA-TA-0.5/LiCl-1 hydrogel stretched to 120% strain at different cycles.

Figure S3. Relative resistance change of PVA-TA-0.5/LiCl-1 hydrogel strain sensors under 120% strain.

Figure S4. PVA-TA-0.5/LiCl-1 hydrogel did not cause inflammation on the skin after 3 hours.

Figure S5. Water retention performance of PVA-TA-0.5/LiCl-1 hydrogel and PVA-TA-0.5 hydrogel.

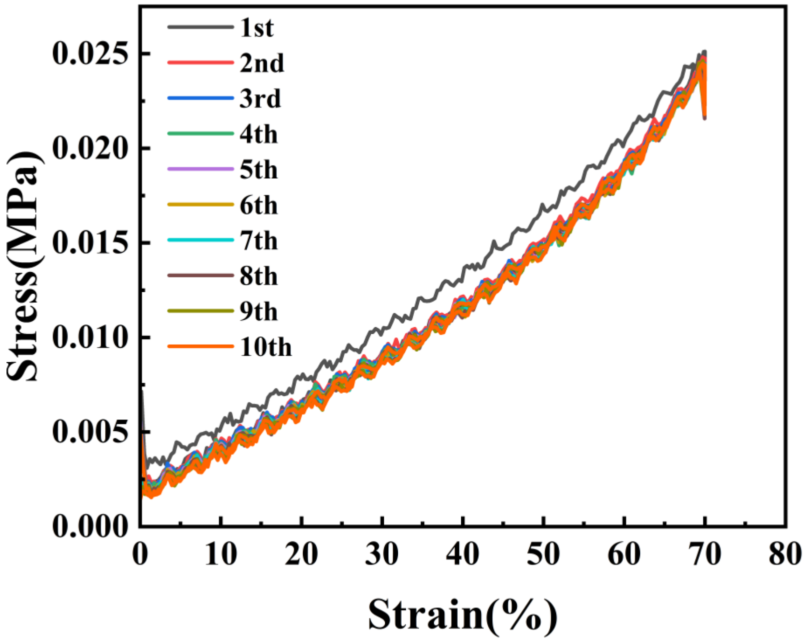

Figure S1. 10 cyclic tensile curves of the PVA-TA-0.5/LiCl-1 hydrogel at the 70% strain.

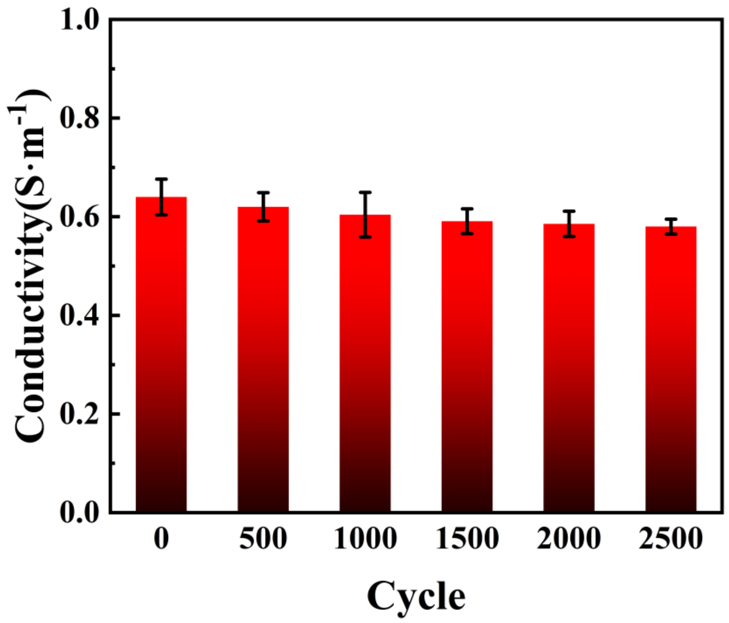

Figure S2. Conductivity of PVA-TA-0.5/LiCl-1 hydrogel stretched to 120% strain at different cycles.

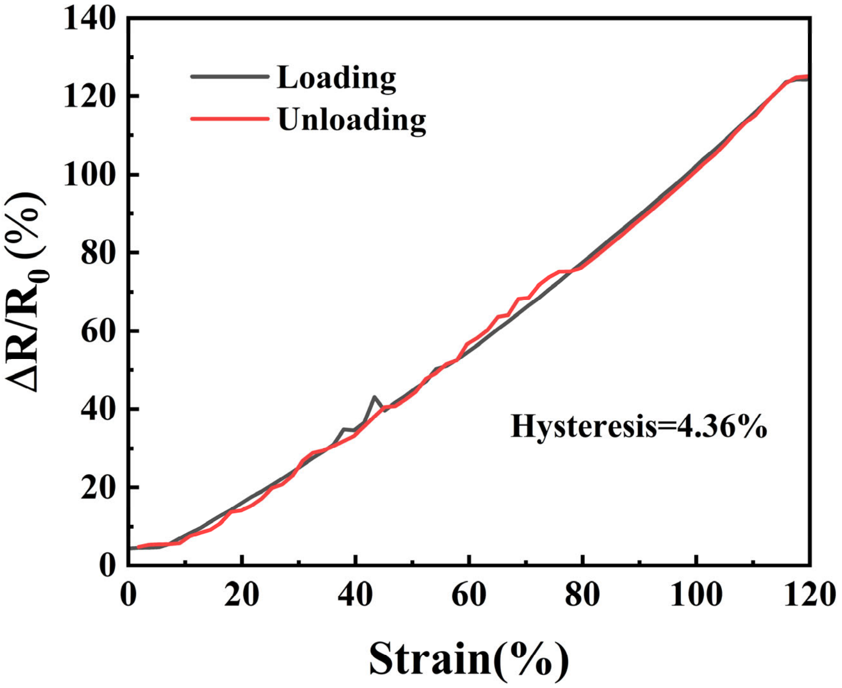

Figure S3. Relative resistance change of PVA-TA-0.5/LiCl-1 hydrogel strain sensors under 120% strain.

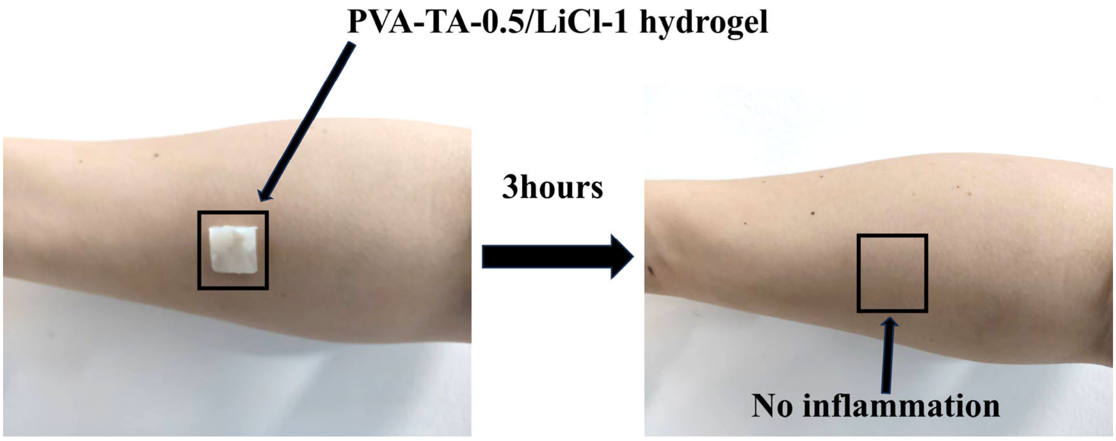

Figure S4. PVA-TA-0.5/LiCl-1 hydrogel did not cause inflammation on the skin after 3 hours.

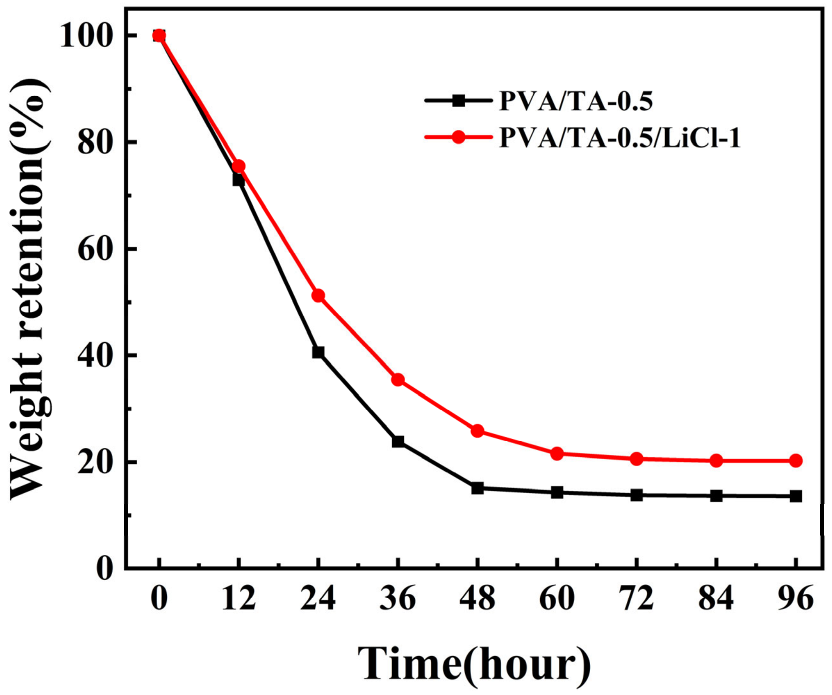

Figure S5. Water retention performance of PVA-TA-0.5/LiCl-1 hydrogel and PVA-TA-0.5 hydroge.
